# “Uremic Toxin” Section in the Journal *Toxins*: A Powerful Tool to Bundle and Advance Knowledge on Uremia

**DOI:** 10.3390/toxins9050170

**Published:** 2017-05-18

**Authors:** Raymond Vanholder

**Affiliations:** Nephrology Section, 0K12, Department of Internal Medicine, Ghent University Hospital, De Pintelaan 185, 9000 Ghent, Belgium; raymond.vanholder@ugent.be

In the journal *Toxins*, a new section has been added to those already included, which will be specifically devoted to uremic toxins. The term uremia refers to the gradual endogenous intoxication that develops with the functional failure of the kidneys, which like the liver and the lungs, are tasked with eliminating useless metabolic waste from the body. The name uremia is derived from the highest concentrated and also first identified uremic retention solute: urea [1]. Kidney failure may develop either in a short time span (Acute Kidney Injury—AKI) or more gradually (Chronic Kidney Disease—CKD). Compounds that, in both conditions, accumulate in the body due to inadequate renal clearance are called uremic retention solutes, and are designated as uremic toxins if they exert a biological effect (toxicity) [2]. Both AKI and CKD affect a substantial proportion of the population and are linked to important morbidity and mortality [3,4]. Since uremic toxins as well as the uremic condition have been shown to affect most of the body functions, it is likely that uremic retention plays a fundamental role in this morbidity and mortality [5]. 

The decision to add this new section on uremic toxins was largely influenced by the success of two Special Issues on this topic that appeared in this same journal: one launched in November 2013 and edited by J Jankowski, and one launched in October 2016 and edited by myself. 

Traditionally, the journal *Toxins* focuses on issues that are related to classical toxicology, which essentially concentrates on exogenous compounds that enter the body from the external environment, e.g., snake or spider venoms, food poisoning, or bacterial and environmental toxins. With uremic toxicity, the journal moves to another area, the area of endogenously produced toxins, generated by the metabolism of the body or by microbiota present in the body (essentially the intestinal microbiota). Their toxicity is, to a large extent, related to their increased concentration in kidney failure as compared to the normal situation, because of their decreased renal clearance, so that solutes which are normally secreted by the healthy kidneys are retained in the body. 

The research and literature on uremic toxicity have been booming in the recent years. Taking indoxyl sulfate as an example, one of the relative newcomers to be recognized as a uremic toxin, this compound was not once the topic of a publication in the years 1970 and 1980, only once in the year 1990 and four times in the year 2000, but 24 times in 2010 and 53 times in 2015. Uremic toxin research has led to the identification of a number of newly detected toxins such as p-cresylsulfate [6], fibroblast growth factor-23 (FGF-23) [7] and trimethylamine-N-oxide (TMAO) [8], but also to the rehabilitation of the status of uremic toxin of solutes that previously had been considered inert and that now appear to exert toxicity, such as urea [9] and symmetric dimethylarginine (SMDA) [10].

It is no surprise that with the extension of our knowledge on uremic toxins and their biological functions, the number of publications on this issue has also grown. These include reports on newly detected toxins and on a broad array of biological effects, especially those leading to cardio-vascular damage, fibrosis and progression of kidney disease, which remain the main killers of the uremic population, but also on strategies to decrease the concentration of uremic toxins and their kinetics during these processes. This can also include other aspects such as the pharmaceutical neutralization of negative biological effects; the enhancement of renal and tubular removal of uremic toxins; extracorporeal removal by adsorption or bio-artificial systems; and the modification of intestinal generation and adsorption [11]. Rapidly developing novel methodological approaches such as those related to metabolomics, proteomics, genomics and transcriptomics, as well as the endlessly growing possibilities of molecular biology can be considered as powerful tools to extend this knowledge which, by the application of biostatistics and innovative acquisitions such as systems biology, may lead to novel therapeutics, and personalized medicine [12].

In addition to further exploration and extension of the knowledge on the effect and removal of uremic toxins, we hope that this section will open avenues to at present insufficiently explored areas of uremic toxicity, such as diet and uremic toxins; modifications of the intestinal milieu and uremic toxin generation; other non-extracorporeal methods to reduce uremic toxin concentration or their effects; uremic toxin retention in Acute Kidney Injury and after kidney transplantation; and the effect of peritoneal dialysis on uremic toxin removal. Furthermore, there is an absolute need for studies in quasi non-existing areas such as Randomized Controlled Trials exploring the effect of decreasing the concentration of specific uremic toxins or groups of uremic toxins on hard outcomes, more novel compact methods for extracorporeal removal allowing more flexibility and quality of life to the uremic patient, and the role of “uremic toxins” in the population without kidney failure, which, if such studies would turn out positive, would allow those molecules to be classified as general, not only uremic, toxins. 

Although, to date, such data could be submitted and still can be submitted to a broad array of journals, it is a positive evolution that from now on a new alternative is offered allowing such articles to be assembled as a group in a specific section of an easily accessible journal dedicated solely to toxicity. We can only hope that our call is followed by a satisfactory number of relevant submissions. 
